# The Outcome of Molecularly Targeted Therapy after Surgical Treatment of Spinal Metastasis

**DOI:** 10.3390/jcm12123920

**Published:** 2023-06-08

**Authors:** Shurei Sugita, Sawako Ogiso, Masanori Fujiwara, Euan Morita, Takuma Koyama, Takahiro Hozumi

**Affiliations:** Department of Orthopaedic Surgery and Musculoskeletal Oncology, Tokyo Metropolitan Cancer and Infectious Diseases Center, Komagome Hospital, Tokyo 113-8677, Japan

**Keywords:** molecular targeted therapy, spinal metastasis, local recurrence, surgery

## Abstract

The aim of this study was to investigate outcomes of molecularly targeted therapy after surgical treatment of spinal metastasis. Participants comprised 164 patients who underwent surgical treatment of spinal metastasis, divided according to whether molecularly targeted therapy was performed. We compared survival, local recurrence of metastasis detected by imaging, the disease-free interval, relapses of neurological deterioration, and the ability to walk between groups. Molecularly targeted drugs were administered to 39 patients after surgery (TT group) and were not administered to 125 patients (non-TT group). Median survival was significantly longer in the TT group (1027 days) than in the non-TT group (439 days, *p* < 0.01). Local recurrence occurred in 25 patients in the non-TT group and 10 patients in the TT group. The disease-free interval did not differ between groups. Neurological deterioration was observed in three patients in the non-TT group and no patients in the TT group. The ability to walk was preserved in 97.6% of patients in the TT group and 88% of patients in the non-TT group (*p* = 0.12). In conclusion, molecularly targeted drugs improve survival in patients with spinal metastasis but do not alter local control of metastatic tumors.

## 1. Introduction

Metastatic spinal disease is a clinically challenging condition affecting approximately 30% of all cancer patients [1,2]. Of those in whom a spinal metastasis develops, 5–10% experience symptoms during the clinical course [3,4]. The natural course of patients with metastatic spinal disease is unfavorable. Untreated patients can become para- or tetraplegic. The clinical burden of this condition is substantial. These conditions are expected to increase in number due to longer life expectancy as a result of continuing advances in oncological therapy.

In particular, the introduction of molecularly targeted therapy (MTT) has dramatically improved patients’ prognoses [5,6,7]. Additionally, there exists a report indicating that individuals afflicted with spinal metastasis may achieve a more favorable prognosis if they are candidates for MTT as a form of treatment [8]. Patients with a spinal metastasis of renal cell carcinoma treated with radiotherapy and MTT showed a higher local progression-free rate than patients who did not receive MTT [9], suggesting that MTT improves not only life expectancy but also local control of spinal metastases. But the impact of MTT on spinal metastases for other tumor types remains uncertain, necessitating further investigation. Our supposition postulates that MTT confers a beneficial influence on local regulation in other tumor types.

The combination of surgery and radiotherapy has been considered standard treatment for a spinal metastasis [10]. However, the complication rate for patients undergoing surgery for spinal metastases is reported to be 20% to 30% [11]. It would be desirable for MTT to be utilized to circumvent a greater number of surgical procedures with high complication rates.

The aim of this present study was to clarify the outcomes of MTT after surgical treatment of a spinal metastasis, especially from the viewpoints of life prognosis and local control.

## 2. Materials and Methods

The participants were 164 patients who underwent surgery for a spinal metastasis at our hospital between January 2004 and December 2017. All the patients received posterior fusion surgery (with or without decompression) and radiotherapy for their spinal metastasis. Radiotherapy was performed intraoperatively with a dosage of 20 Gy [12]. The patients were grouped into those who received postoperative MTT (the TT group) and those who did not (the non-TT group), and survival, local metastasis recurrence on imaging studies, the disease-free interval, neurological deterioration (in the first three months), denosumab or bisphosphonate administration during treatment, and the ability to walk were compared between groups. Differences in postoperative drug treatment were examined retrospectively according to the medical records. The MTT did not contain the usage of denosumab or bisphosphonate. MTT exclusively pertains to the utilization of anticancer agents. Magnetic resonance imaging (MRI) was performed to screen for local metastases in all the patients every three months. A local recurrence was identified on imaging studies by radiologists. The disease-free state was defined as the absence of any obvious local recurrence at the metastatic site.

All statistical analyses were performed using EZR (Saitama Medical Center, Jichi Medical University, Saitama, Japan), a graphical user interface for R (The R Foundation for Statistical Computing, Vienna, Austria). More precisely, EZR is a modified version of R Commander designed to add statistical functions frequently used in biostatistics [13]. Descriptive statistics were used to analyze the clinical and test data and demographic factors. Continuous variables were expressed as the median and interquartile range (IQR). Differences between groups were compared using Student’s *t*-test for continuous variables and the chi-square test for categorical variables, as appropriate; *p* < 0.05 was considered to indicate statistical significance.

## 3. Results

Table 1 shows the patients’ demographic data. Almost half (44%) the patients were in a non-ambulatory state at the time of surgery. MTT was provided to 39 patients postoperatively, while the remaining 125 patients did not receive MTT. No significant difference was noted between the two groups in terms of age, sex, walking ability, or life expectancy [14]. The ratio of the administration of skeletal-related event prophylactic agents, like zoledronic acid and denosumab, was higher with statistical significance within the TT group.

**Figure 1:**
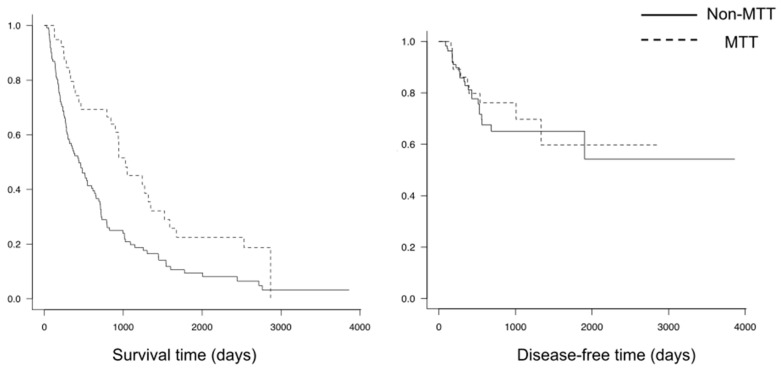
Overall survival and disease-free survival in the two groups.

The median period of survival was significantly longer in the TT group (1027 days) than in the non-TT group (439 days, *p* < 0.01) (Figure 1, Table 2). A local recurrence occurred in 25 patients in the non-TT group and in ten patients in the TT group (*p* = 0.5). The disease-free interval did not differ significantly between the groups, with both groups showing relatively good local control (Figure 1). Neurological deterioration was observed in three patients in the non-TT group and in none of the patients in the TT group (*p* = 1.0). In total, 97.6% of the patients in the TT group and 88% in the non-TT group retained or regained the ability to walk (Table 2).

**Figure 2:**
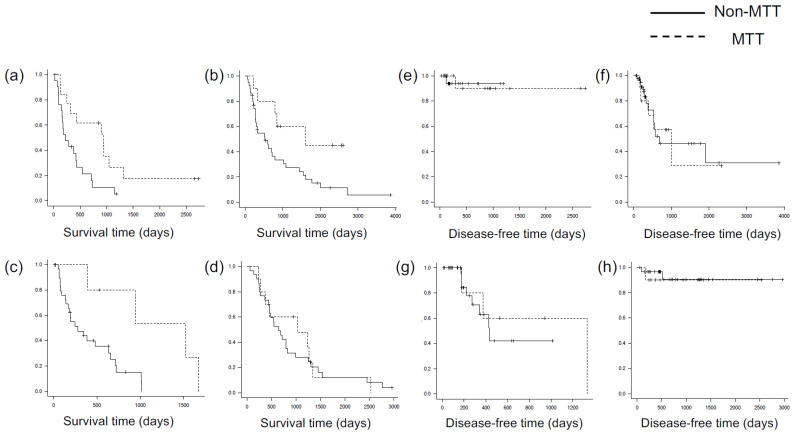
Overall survival for each type of primary tumor: (**a**) lung; (**b**) renal; (**c**) colon; (**d**) breast. Disease-free survival for each type of primary tumor: (**e**) lung; (**f**) renal; (**g**) colon; (**h**) breast. + stands for censoring.

A subgroup analysis was done to compare overall survival and disease-free survival by primary tumor type. Survival time was significantly longer in patients with lung cancer, renal cell carcinoma, and colon cancer with MTT, but no significant difference was seen for breast cancer (Figure 2, Table 3). Disease-free survival tended to be longer in patients with renal cell carcinoma and colon cancer, but the difference was likewise not significant (Figure 2, Table 3). Table 3 shows the one-year disease-free rate. All types of cancer were relatively well-controlled in both groups except for colon cancer in the non-TT group (Figure 2).

## 4. Discussion

In the present study, the life expectancy of patients with a spinal metastasis was longer with MTT than without MTT. However, local control of spinal metastasis did not differ between the groups, with both groups showing relatively good local control.

In general, the life expectancy of patients with a spinal metastasis has significantly improved with the emergence of MTT [5,6]. A recent study demonstrated that local control of a spinal metastasis of renal cell carcinoma also improved with MTT [9]. Among patients with renal cell carcinoma, the combination of MTT and surgery for a bone metastasis significantly improved overall survival [8,15,16]. Our study further supported the existing data suggesting that MTT conferred significantly longer survival on patients who received the therapy than on those who did not. However, with respect to the local control of metastases, no significant difference was observed in the local recurrence rate or disease-free survival rate, even in renal cell carcinoma cases (Table 3). One possible explanation for this finding is the presence or absence of surgical treatment. In the previous study cited above [9], all the patients received radiotherapy alone for their metastasis. In the present study, however, all the patients were treated using a combination of surgery and radiotherapy. The discrepancy between the previously reported results and our own findings may be explained if the contribution of surgery was large enough to diminish the effect of the MTT. Renal cell carcinoma is considered to be a radioresistant tumor, so the patient demographic not receiving surgical intervention may have exhibited a greater likelihood to derive advantage from pharmaceutical intervention. If this suggestion is true, a similar logic might also be thought to apply to the administration of denosumab and bisphosphonate, which were frequently given to the patients in the TT group in the present study. The administration of these medications may have contributed to an enhanced modulation of spinal metastasis local control within the treated cohort. Nevertheless, there was no statistically significant variance in the local control of spinal metastasis between the TT group and the non-TT group. These results may also suggest that the effects of surgery outweighed those of drug therapy, such as denosumab and bisphosphonate.

The combination of surgery and radiotherapy has been considered standard treatment for a spinal metastasis [10]. In our study, MTT did not significantly alter the local control of spinal metastasis in comparison with the control group despite most patients achieving rather good local control and a favorable neurological prognosis. It remains unclear as to whether MTT can stop or delay the progression of bone metastasis besides cases of renal cell carcinoma, as reported in a previous study [9]. Although no definitive conclusions can be drawn from the findings of the present study, in spinal metastases with Bilsky grade [17] two or higher, surgical intervention should be considered because the effects of MTT on local metastases are uncertain. Administration of MTT as an alternative to surgical intervention for spinal metastases is not recommended.

The present study has several limitations. First, MTT administration was not randomly assigned. Instead, allocation depended on the general status and genetic type of the carcinoma in each patient, thus introducing selection bias. However, no significant difference in preoperative neurological status or life expectancy was evident between the groups (Table 2). Second, there exists a possibility that undisclosed confounding variables are exerting influence, given the diversity of patient background and treatment content, as well as the lack of standardization in the background. Third, no significant change in disease-free survival was evident between the groups. However, the median disease-free survival time was longer for patients with renal cell carcinoma and colon carcinoma. For lung and breast cancers, the median values were unable to be calculated because of the relatively good local control achieved. If the observational period had been longer, or if more patients had been enrolled, the results might have been more definitive. Fourth, all the patients underwent surgery for their spinal metastasis. The significance of surgery on the outcomes was therefore unable to be accurately assessed. A similar comparative analysis is required amongst a cohort of patients who were not subjected to surgical intervention for spinal metastases. 

Based on these findings, we concluded that MTT was found to improve survival in patients with a spinal metastasis but did not alter local control of metastatic tumors. Administration of MTT as an alternative to surgical intervention for spinal metastases is not recommended.

## Figures and Tables

**Table 1:** Comparison of background characteristics.

	TT Group	Non-TT Group	*p* Value
No. of patients	39	125	
Age (years)	61 (53–65)	61 (54–68)	0.39
Sex (male:female)	17:22	74:51	0.1
Ambulatory (yes/no)	21/18	74/51	0.58
Frankel scale (*n*)			
A	0	0	0.892
B	1	3	
C	18	51	
D	12	38	
E	8	33	
New Katagiri’s score			
0–3	23	55	0.287
4–6	13	57	
7–10	3	13	
Use of denosmab/BP (yes/no)	22:18	41:84	<0.05

Values represent median (IQR) or number of patients.

**Table 2:** Comparison of clinical outcomes.

	TT Group	Non-TT Group	*p* Value
Survival time (days)	1027	439	<0.05
Local recurrence (*n* (%))	10/40 (25%)	25/125 (20%)	0.5
Disease-free interval (days)	N/A	N/A	0.59
One-year disease-free rate (%)	86.1	82.8	
Walking ability (yes/no (%))	39/1 (97.5%)	110/15 (88%)	0.12
Neurological deterioration (*n*)	0 (0%)	3 (2.4%)	1

N/A = not applicable.

**Table 3:** Comparison of clinical outcomes in each primary tumor.

	TT Group	Non-TT Group	*p* Value
Lung carcinoma	14	22	
Survival time (days)	902	219	<0.05
Disease-free interval (days)	N/A	N/A	0.98
One-year disease-free rate	90.9	93.8	
Renal cell carcinoma	10	39	
Survival time (days)	1590	518	<0.05
Disease-free interval (days)	1008	684	0.97
One-year disease-free rate	80.0	78.0	
Colon carcinoma	5	31	
Survival time (days)	1523	281	<0.05
Disease-free interval (days)	1333	431	0.62
One-year disease-free rate	80.0	62.8	
Breast carcinoma	10	30	
Survival time (days)	1027	649	0.75
Disease-free interval (days)	N/A	N/A	0.76
One-year disease-free rate	90.0	96.6	

N/A = not applicable.

## Data Availability

Data is unavailable due to privacy restriction.
